# The Influence of Large-Scale Airborne Particle Decline and Traffic-Related Exposure on Children’s Lung Function

**DOI:** 10.1289/ehp.8180

**Published:** 2005-09-20

**Authors:** Dorothea Sugiri, Ulrich Ranft, Tamara Schikowski, Ursula Krämer

**Affiliations:** Institut für umweltmedizinische Forschung an der Heinrich-Heine-Universität Düsseldorf, Düsseldorf, Germany

**Keywords:** air pollution, children, German reunification, lung function, particulate matter, repeated cross sections, traffic

## Abstract

Between 1991 and 2000, ambient air pollution in East Germany changed to resemble West German pollution levels: The concentration of total suspended particles (TSPs) decreased on a broad scale while traffic increased. During that time, we analyzed total lung capacity (TLC) and airway resistance (*R*_aw_) of East and West German children. We tested children 5–7 years of age (*n* = 2,574) with cooperation-independent body plethysmography in repeated cross sections. We used random-effect models to determine the mutually adjusted association between lung function and short-term and chronic particle exposure and its interaction with living near a busy road. Annual averages of TSPs declined from 77 to 44 μg/m^3^; averages on the day of investigation declined from 133 to 30 μg/m^3^. Differences in lung function between East and West German children vanished during the investigation time. The association of TSPs with *R*_aw_ and TLC was stronger in children living > 50 m away from busy roads. East German children from this group had an *R*_aw_ 2.5% higher [95% confidence interval (CI), 0.0–5.1%] per 40-μg/m^3^ increase of daily TSP averages. TLC decreased by 6.2% (95% CI, 0.04–11.6%) per 40-μg/m^3^ increase in annual mean TSPs, and this effect was equally pronounced in East and West Germany. TSP exposure decreased on a broad scale between 1991 and 2000. Lower concentrations of TSPs were associated with better measures of lung function in 6-year-old children. For children living near busy roads, this effect was diminished.

Before the reunification of Germany in 1989, outdoor mass concentration of total suspended particles (TSPs) was higher in East Germany than in West Germany because of emissions from industry and domestic sources, but traffic-related air pollution was higher in West Germany than in East Germany. During the first years after reunification, TSP levels declined considerably in East Germany and reached the West German levels, but emissions from traffic increased. The size distribution of airborne particles exhibited a shift toward finer particles (Ebelt et al. 2001; Kreyling et al. 2003). The effect of this changing pattern on lung function in 6-year-old children has not been investigated so far.

To date, evidence for an effect of ambient air pollution exposure on lung function in children comes from studies investigating school children at least 8 years of age. All these studies used spirometric measures [forced vital expiratory capacity (FVC), forced expiratory volume in 1 sec (FEV_1_), and peak expiratory flow (PEF)], which depends on cooperation of the children and thus is more difficult to manage in children of a younger age. Most studies, with one exception (Dockery et al. 1989), found an adverse association with chronic exposure to air pollution (Islam and Schlipköter 1993; Peters et al. 1999; Pope and Arden 2000; Schindl 1993; Schwartz 1989). Elevated levels of urban ambient air pollution were found to retard development of children’s lung function (He et al. 1993; Gauderman et al. 2000, 2002, 2004). Decline of long-term exposure to air pollution was associated with an increase of FVC (Frye et al. 2003). Lung function of children has been associated with traffic-related exposure in a number of epidemiologic studies (Brunekreef et al. 1997; Fritz and Herbarth 2001; Wjst et al. 1993). Acute effects were mostly investigated independently from long-term effects. Short-term ambient exposure of children to fine particles (Hoek et al. 1998) or winter air pollution (Peacock et al. 2003) resulted in PEF decrement and small but statistically significant decline in forced expiratory volume in 0.75 sec and FEV_1_ (Pope and Arden 2000). Adults also showed decreased forced expiratory volumes and flows (Schindler et al. 2001).

In this study we added four new aspects to the body of evidence concerning the association between outdoor air pollution and lung function in children. First, in 6-year-old children, younger than in most previous studies, we investigated the association of lung function with TSPs and traffic-related pollution. Young children are a susceptible subgroup for air pollution health effects (Kim 2004; Mathieu-Nolf 2002). We targeted 6-year-old children because the adolescent spurt has not started yet (Wang et al. 1993a, 1993b). Because of the young age of the subjects, we used measures of lung function that require little collaboration [airway resistance (*R*_aw_) and total lung capacity (TLC)].

Second, we simultaneously considered the influence of short-term and chronic TSP exposure on lung function. *R*_aw_ reacts rapidly to short-term exposure by means of bronchoconstriction and/or hypersecretion and might therefore preferentially show acute effects, whereas TLC changes slowly and might show long-term effects. Third, we compared effects of TSP exposure on lung function in children from East and West Germany, thereby adjusting for many additional changes in lifestyle that occurred between 1991 and 1997 in East Germany after reunification. Fourth, the effects of traffic-related exposure were included in our study. We expected the effects of living near a busy road to increase between 1991 and 1997 in East Germany, thereby diminishing the effects of the broad-scale decrease in TSPs.

## Materials and Methods

### Study subjects and sites.

This study is part of a large study in East and West Germany investigating the health effects of the changing environmental and socioeconomic conditions after reunification in school beginners (6 years of age) between 1991 and 2000 (Krämer et al. 1999, 2002). Every school beginner from preselected geographical areas was invited to participate in a questionnaire investigation, and every second child was asked to do lung function measurements. Rural areas without heavy industrial impact were Salzwedel and Osterburg in East Germany and Borken in West Germany; urban areas with industrial impact were Halle-Centre, Leipzig-Southwest, and Magdeburg-Centre in East Germany and Duisburg-North and Duisburg-South in West Germany. Essen-Centre and Cologne-Centre in West Germany were urban areas with strong traffic burden. Lung function was recorded in East Germany in 1991, 1994, and 1997 and in West Germany in 1991, 1994, 1997, and 2000.

### Study design.

Consecutive cross-sections of school beginners were investigated to estimate the effect of outdoor pollution with TSPs and living near a road with heavy traffic on lung function. Measurements of lung function were *R*_aw_ and TLC. Covariates were age, sex, height, body mass index (BMI) > 18.4 kg/m^2^ (95th percentile of BMI of study children from East German urban areas in 1991), birth weight < 2,400 g (5th percentile of birth weights of study children from East German urban areas in 1991), parental education [highest achieved grade of schooling of either parent; we distinguished two grades, ≤ 10th grade (American grade “high school”) and > 10 years of schooling (American grade “college and higher degrees”)], bedroom sharing (one or more people share the child’s bedroom), single room heating with fossil fuels, cooking with gas, tobacco smoke exposure considering passive smoking at home and/or maternal smoking in pregnancy, and outdoor temperature < 0°C at the day of investigation.

### Lung function.

We tested lung function with a constant volume body plethysmograph apparatus from Jaeger (Würzburg, Germany). Principles of this technique were summarized by Ulmer et al. (1991). Testing was done with the same mobile body plethysmograph and the same experienced team of examiners in all regions and over all the years of study. The lung function of children suffering from acute airway infection was not recorded because this could confound the results. The investigations were conducted in early spring in East Germany and in late spring in West Germany. Several tests on a child were performed for each respiratory maneuver. Registering began with normal respiration for determining *R*_aw_, followed by maximal expiration and maximal slow inspiration for determining TLC. This method of measurement is precisely described by Coates et al. (1997). We used the child’s mean values for *R*_aw_ for analysis; however, only observations of those children where the measured values of *R*_aw_ varied < 0.3 kPa-sec/L were included. The chosen value of TLC was the maximum of valid single measurements. Valid measurements of TLC were those where the simultaneously registered inspiratory capacity was within 80–140% of the child’s averaged intrathoracic gas volume.

### Exposure.

Daily and annual mean values of TSPs and sulfur dioxide were determined by the regional authorities of Northrhine Westfalia (West German study sites) [Landesamt für Immissionsschutz des Landes Nordrhein-Westfalen (LUA) 1990], Saxony (Leipzig) (Bezirkshygieneinstitut Leipzig 1990), and Saxony-Anhalt (East German study sites except Leipzig) (Bezirkshygieneinstitut Magdeburg 1990) by the same methods: radiometric technique (β-ray absorption monitor) for determination of TSPs and ultraviolet fluorescence method for determination of SO_2_. Children’s exposure to outdoor TSPs and SO_2_ was characterized by the mean of the values gained at the monitoring station(s) (1–3) in the investigation areas. These were either mean values of the year before the study (chronic) or the mean values of the investigation day (short-term). The measurement stations for TSPs and SO_2_ were built to represent urban or rural large-scale background exposures. Therefore, they are situated far from industrial sources or roads with heavy traffic. These stations may not adequately describe exposure of children who live near roads.

Parents were asked “How far away is your address (beeline) from a busy street (rush hour traffic/through traffic),” and answer categories were predefined as “< 50 m” and “> 50 m.” We used this information to define two categories (high and low) of traffic exposure. This assessment was validated in the group of children from West Germany in 2000 where geocoded addresses and a road network with traffic density data were available. We calculated daily traffic flow within a circular neighborhood of 50 m radius around a child’s address as the sum of all products of number of cars per day times the length of its street section for all segments of this network located within the circle. These traffic densities data were compared for children with high and with low traffic exposure as determined by questionnaire assessment.

The outdoor temperature was measured during the day of investigation at the place of the mobile body plethysmograph and included in the analysis as daily mean temperature.

### Questionnaire.

We derived covariates from a questionnaire, which was sent to the parents along with the invitation to the school entrance examination, which is compulsory in Germany. On the day of investigation, the questionnaire was checked by physicians from the local health authorities and subsequently completed by the parents.

Height and weight of the children were measured using standardized procedures by the assistants of the local health authorities.

The ethical committee of the Medical Association of Saxony Anhalt approved the study. Written informed consent was obtained from the parents.

### Statistical analyses.

Only children with German nationality living > 2 years at their residence were included. Furthermore, asthmatic children were excluded to evade effects of broncholytic medication.

All analyses were done by linear regression on logarithmically transformed lung function measurements. All covariates mentioned above were included in the regression models. We tested homogeneity of TSP effects on children from East and West Germany as well as on children living in proximity to and at a distance from busy streets by including product terms. We also tested the TSP effects on TLC and *R*_aw_ by including a linear term for trend (study year). The estimated parameters of the regression models were expressed as geometric means ratios (MRs) of the respective lung function variable for an increment of one unit of continuous variables or for 1 versus 0 of binary variables, adjusted for the remaining covariates. The unit for TSPs was 40 μg/m^3^, which is the span between the 5th and 95th percentile of occurring annual mean values. Possible clustering by area was accounted for by random effect modeling.

All statistical analyses were done with SAS (version 8.2 for Windows NT; SAS Institute Inc., Cary, NC, USA). Regression models were computed with the procedure GENMOD (SAS Institute Inc.).

## Results

### Response.

Lung function data were available for 3,540 children (response, 69% in West and 73% in East Germany). Children of non-German nationality (*n* = 596), children living < 2 years at their place of residence (*n* = 401), and children suffering from asthma (*n* = 64) were excluded from the analysis. This resulted in 2,574 children with valid data for *R*_aw_ and 2,066 children with valid data for TLC. Three percent of the children had no valid measurement for *R*_aw_, and 22% had no valid measurement of TLC. No lung function differences were found between children with and without valid TLC measurement. Both groups showed identical mean *R*_aw_ of 0.63 kPa × sec/L. The lower rate of valid values for TLC compared with *R*_aw_ is plausible, because TLC measurement requires some motivation for a respiratory maneuver, whereas *R*_aw_ is virtually independent from collaboration. The final group for analysis consisted of 2,275 children where information on all covariates was available.

### Exposure.

Table 1 shows that TSP and SO_2_ concentrations in outdoor air decreased mainly in East Germany; however, a slight decrease was also found in West Germany. When the study started in 1991, the annual mean TSP concentrations in East Germany exceeded West German concentrations by a factor up to 1.5, and the daily mean concentrations were up to three times higher in East than in West Germany. Already in 1994, annual TSP levels were similar in East and West Germany. The annual and daily mean TSP concentrations were moderately correlated (*r* = 0.68). Autocorrelation of TSP concentration values at the day of investigation compared with the day before was *r* = 0.83. Differences in SO_2_ between East and West Germany and the decrease of SO_2_ in East Germany were much stronger than for TSPs: The annual mean SO_2_ concentrations in East Germany exceeded West German concentrations by a factor up to 5, and the daily mean concentrations were > 10 times higher in East than in West Germany. SO_2_ and TSPs were highly correlated. Annual means showed a correlation coefficient of 0.82 and daily means of 0.82. Since 1997, annual SO_2_ levels were similar in East and West. The annual and daily mean SO_2_ concentrations were more strongly correlated (*r* = 0.91) than TSP concentrations. Autocorrelation of SO_2_ concentration values at the day of investigation compared with the day before was *r* = 0.91.

Parent’s judgment about distance to a busy street (Table 2) was tested by comparing the two exposure groups with respect to objective measure of traffic density in the subsample of children in West Germany 2000, where geocoded residential addresses and data of traffic density were available. Mean traffic density within 50 m radius around the children’s addresses of those claiming high traffic exposure was 216 traveled km/day [no significant differences between the rural Borken (241 km/day) and the urban Duisburg (204 km/day)], and for those claiming low traffic exposure it was 34 traveled km/day (26 in Borken and 41 in Duisburg). The difference between means of traffic exposure groups was highly significant (Wilcoxon test, *p* < 0.000001).

### Covariates.

A description of covariates and traffic exposure is given in Table 2. School beginners were slightly younger in East than in West Germany because of the earlier examination phase in East Germany. The prevalence of the adverse lifestyle factors: heating with fossil fuels, gas for cooking, and environmental tobacco smoke, respectively, decreased strongly in East and slightly in West Germany since 1991. Low birth weight decreased and BMI increased in East as well as in West Germany. In West Germany, parental educational level seemed to be lower in 2000 than in 1991, perhaps because of the selection of different study subareas in the West German study areas. Some East German school beginners in 1991 and 1994 were investigated while outdoor temperatures were < 0°C, which can elevate *R*_aw_.

### Association of TSPs and distance to a busy road with lung function.

Table 3 shows mean crude lung function parameters grouped by study year and part of Germany. The results of the regression analysis are presented in Table 4. The interaction between annual mean TSPs and traffic exposure on TLC was significant (MR = 1.02 for TSP effect living near a busy road/TSP effect living far from a busy road, *p* = 0.0004), indicating that the decrease of TLC per unit TSPs was smaller for children living near a busy road than for those living far away from it. The interaction between daily mean TSPs and traffic exposure on *R*_aw_ was also significant (MR = 0.98, *p* < 0.0001). The interaction between TSPs and region (East/West) on TLC was not significant, indicating that the TSP effect on TLC was the same in East and in West Germany. This interaction, however, was significant for *R*_aw_. To facilitate interpretation, the results are also presented stratified by region (East/West) and distance from a busy road in Table 5.

In children living farther away from a busy road, *R*_aw_ showed a positive association with short-term TSP exposure (mean on the day of investigation) for children from East Germany but no association for children from West Germany. Mutual adjusting of annual TSPs with annual SO_2_ and daily TSPs with daily SO_2_ resulted in loss of significance and a paradoxical direction of short-term TSP effect on *R*_aw_ in East Germany (data not shown).

**Figure 1:**
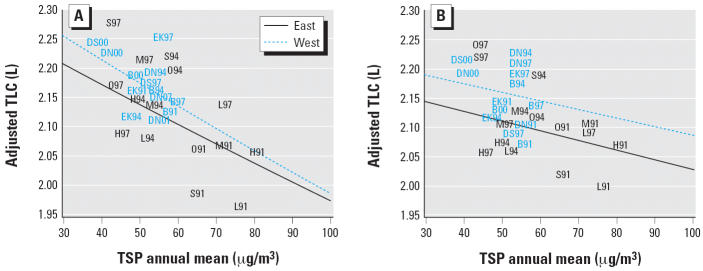
TLC of German children living > 50 m (*A*) and ≤ 50 m (*B*) away from a street with heavy traffic, living at least 2 years at their place of residence and without asthma: place- and year-specific geometric mean values and regression line adjusted for education, bedroom sharing, sex, age, height, BMI, birth weight, heating with fossil fuels, cooking with gas, passive smoking and/or maternal smoking during pregnancy, and outdoor temperature on the day of investigation. Data points indicate place and year of investigation: B, Borken; DN, Duisburg north; DS, Duisburg south; EK, Essen and Cologne; H, Halle; L, Leipzig; M, Magdeburg; O, Osterburg; S, Salzwedel.

TLC was about 3% smaller for children living in residential areas where TSP annual means were increased by 40 μg/m^3^ when they additionally lived near a busy street. This result was not significant. However, children dwelling far from busy roads had a TLC reading > 6% lower when their home was located in a region where TSP annual means were increased by 40 μg/m^3^. Figure 1 represents this result graphically. Comparing Figure 1A and 1B shows that the effect of broad-scale TSPs on TLC is stronger when only considering children living far from a busy road. The regression coefficients (Table 5) were similar in East and West Germany. The effects did not change when not mutually adjusting for short-term TSPs. An additional effect of short-term exposure on TLC could not be detected. The chronic TSP effect on TLC scarcely differed when adjusting for annual SO_2_ (data not shown).

An overall effect of living near a busy road on *R*_aw_ and TLC could be detected in West Germany. In East Germany, this effect emerged only when restricting the analysis to children investigated from 1994 onward (Table 6).

### Sensitivity analysis.

We excluded asthmatics because medication use could confound both the individual observations and the changing trends over time. On the other hand, children with asthma might be more susceptible to air pollution exposures. We therefore repeated the analysis without excluding asthmatic children. As found by Gauderman et al. (2004), effects in the total group and in the group excluding children with asthma did not differ substantially. All effects in the total group, however, were less pronounced: the means ratio for the association between TLC and TSPs in East Germany was 0.962 instead of 0.961, and in West Germany was 0.956 instead of 0.953.

Big differences as well as massive changes in indoor combustion sources existed between East and West Germany. In our analyses we adjusted for indoor combustion sources. Additionally, we stratified the data by indoor sources and repeated the analysis for the group without special sources. The results did not change, and all associations between TSPs and lung function stayed significant, when excluding children heating their home with fossil fuels; for example, the long-term effect on TLC only differed by about 1% with respect to the means ratio.

To account for unknown factors changing over time, we additionally adjusted for trend. The effect estimates for TLC and annual mean TSPs in children living > 50 m from a busy road changed from 0.938 to 0.957 but was still significant. The effect estimate for *R*_aw_ and daily TSPs for East German children living > 50 m from a busy road changed from 1.025 to 1.027 and remained significant.

There is evidence to suggest that short-term effects of particulate matter (PM) may be strongest 2–5 days after the exposure (Delfino et al. 2004). Therefore, we considered modeling different lags of TSP exposure and included in the analysis TSP values from the day before the investigation or the means from the week before the investigation instead of the values on the day of investigation. All these measures were highly correlated (TSPs on the day of investigation with TSPs the day before, *r* = 0.83; TSPs on the day of investigation with the of the week before, *r* = 0.82), and the effect estimates did not change; that is, all relative differences of means ratios between models of different lags were < 1%.

## Discussion

In 1991, 6-year-old children in East Germany had poorer lung function values than did children in West Germany: *R*_aw_ was higher and TLC lower in East than in West German children. East/West differences in lung function diminished between 1991 and 1997. At the same time, the East/West differences in TSP concentrations (daily and annual) also diminished. Lower concentrations of TSPs were associated with better measure of lung function in 6-year-old children. TLC was mostly affected by chronic exposure and *R*_aw_ by short-term exposure. Traffic-related exposure had a negative impact on lung function, and for children living near roads with heavy traffic, the positive effect of lower TSP concentrations was smaller.

Although young children might be especially vulnerable to air pollution health effects (Kim 2004; Mathieu-Nolf 2002), data on lung function for this group of children are rarely presented because lung function measurements using the usual spirometric device depend on children’s cooperation. We therefore used a mobile body plethysmographic device. The TLC measurements, however, require a small amount of cooperation, which might explain the 22% nonvalid TLC measurements. We have no indication that the observed TLC results were in any way distorted by the lower number of valid measurements. *R*_aw_ in children with and without valid TLC measurements was found to be equal. The overall response rate to our study was reasonable (72%). We assume that our results are not biased by any changes in measuring lung function, because one team examined all the children using the same equipment, same method of measuring, and the same calculations throughout the study period. However, these restrictions had the disadvantage that the investigations in East Germany could not be done simultaneously with those in West Germany; they always preceded them. Because climate and pollen exposure differed between February/March (investigations in East Germany) and April/May (investigations in West Germany), a possible distortion of the East/West German comparison had to be considered. The convergence of mean lung function parameters between 1991 and 1997 is probably not caused by the timing of the studies because in all years the investigations were done in the same months in East and West Germany. Differences in seasonal factors could influence East/West comparisons in lung function over time if they influence lung function and change differently over the investigation years in East and West Germany. Among factors that might have introduced seasonal variation in lung function are outdoor temperature, acute infections, and pollen exposure. Temperature was adjusted for in the present analysis. No additional effects on lung function of outdoor temperature > 0°C could be detected, and the temperature differences between the East and West German investigation times did not change in a systematic way (7.4°C in 1991, 8.9°C in 1995, and 4.7°C in 1997). Children with acute respiratory infections were excluded from the analysis. The birch pollen season was always included in the investigation time in West Germany but never in East Germany. There was a trend to earlier birch pollen seasons; however, the seasonal mean pollen concentration showed no trend (*p* = 0.3), and we could detect no effect of pollen exposure on lung function in nonasthmatic children (*R*_aw_: MR = 0.998, *p* = 0.291; TLC: MR = 0.999, *p* = 0.346). The stronger effect of short-term TSP exposure on *R*_aw_ in East Germany compared with West Germany, however, could be partially because short-term TSP concentrations were higher in early spring (investigation in East Germany) than in late spring (investigation in West Germany).

Measurements of PM_10_ (PM with aerodynamic diameter < 10 μm) or finer particle fractions were not available during the observation period. In Germany, low-volume samplers were used for measuring TSPs. An often used conversion factor (PM_10_/TSPs) is 0.86 (Gehrig and Hofer 2000), which seems to be quite constant even during 1991–2000 in East Germany (Heinrich J, personal communication). When using this factor, our result of 3.9% (East combined) and 4.7% (West combined) lower TLC in children exposed to 40 μg/m^3^ higher TSPs would transform to 4.6 and 5.4% per 40 μg/m^3^ higher PM_10_.

Other air pollutants such as SO_2_, nitrogen dioxide, or ozone might have caused effects similar to those we ascribed to TSPs. NO_2_ or O_3_ measurements were available only from 1992 onward. Short-term SO_2_ concentration changed from 242 to 10 μg/m^3^ during the observation period, and annual means changed from 127 to 20 μg/m^3^. The TSP effects on TLC remained unchanged, whereas the short-term TSP effect on *R*_aw_ lost significance when additionally adjusting for SO_2_. Therefore, short-term TSP effects might be due to a combined action of TSPs and SO_2_.

The TSP effect was significant after adjusting for the covariates as presented in Table 2. However, we may have missed some covariates that changed over time. Therefore, in a sensitivity analysis, we additionally adjusted for trend. The long-term effects on TLC and the short-term effects on *R*_aw_ were statistically significant even after adjusting for trend. Another aspect that adds to the possible causality of TSPs is that the long-term effects on TLC were nearly equally pronounced in East Germany and in West Germany, where lifestyle was different and changes during 1991–2000 were much less pronounced than they were in East Germany.

We found that living next to a busy road had an adverse effect on *R*_aw_ and TLC. This effect emerged in East Germany only after 1991, indicating strengthening over time. We found that the TSP effects were stronger for children living away from roads with heavy traffic. In this group, *R*_aw_ was positively associated with short-term TSP concentrations and TLC with long-term concentrations. After reunification, in East Germany the number of automobiles increased: in Saxony (East Germany), from 1.2 million to 2.1 million [Statistisches Landesamt des Freistaates Sachsen (SLFS) 2000]; in Saxony Anhalt (East Germany), from 0.8 million to 1.2 million [Landesamt für Umweltschutz Sachsen-Anhalt (LAU) 2004]. However, in Northrhine Westphalia (West Germany) the number increased only from 8 million to 9 million [Ministerium für Verkehr, Energie und Landesplanung des Landes Nordrhein-Westfalen (MVEL) 2004]. The number of cars per 1,000 inhabitants changed in East Germany from 245 to 488 between the years 1989 and 1997, resulting in a number nearly equal to that found in West Germany (517). Because of propagation of catalytic converters, PM and nitrogen oxide (NO_x_) emissions from traffic-related sources in East Germany did not increase proportionally to the increase in automobile numbers, but peaked in 1993 (PM) and 1995 (NO_x_) and has since declined in the years thereafter [Landesamt für Umwelt und Geologie, Freistaat Sachsen (LfUG) 1997]. The relative contribution of traffic-related sources to all emissions increased between 1989 and 1997 for PM from 2 to 22% and for NO_x_ from 30 to 48%. A measurement station with traffic exposure in Leipzig, one of our study areas, showed a steady increase of NO_2_ annual means from 39 μg/m^3^ in 1991 to 53 μg/m^3^ in 1996 (LfUG 1997). The number concentration of ultrafine particles (aerodynamic diameter 0.01–0.02 μm) in East Germany increased after 1991 despite decreasing TSP concentrations and decreasing concentrations of fine PM (aerodynamic diameter < 2.5 μm) (Ebelt et al. 2001; Kreyling et al. 2003). Ultrafine particles from automobile emissions vary on a small spatial scale; they disappear exponentially with the distance from a major road and reach background levels at a distance of 300 m (Zhu et al. 2002). Similar effects can be observed for other traffic-related air pollutants. In East Germany, overall background TSP concentrations decreased whereas concentrations of certain traffic-related substances near roads with heavy traffic actually increased. Therefore, it seems plausible that the effects of decreasing TSP concentrations in outdoor air are more pronounced in areas that are farther away from roads with heavy traffic, whereas the effects are possibly counteracted by the increasing concentrations of traffic-related pollutants. We did not measure traffic-related substances, but relied on questionnaires. We were able to show in a small validation study that the questionnaire information about a busy road in 50 m distance seems to be a good indicator for traffic in a 50-m radius around the child’s address. Lung function of children has been found to be associated with traffic-related exposure in a number of other epidemiologic studies (Brunekreef et al. 1997; Fritz and Herbarth 2001; Wjst et al. 1993). Nevertheless, the counteracting effect of living near a busy road might be caused by factors other than air pollution; for example, proximity to a busy street would result in less ability to play outdoors because of high volumes of traffic in front of the home.

We are not aware of any other major epidemiologic study having measured TLC or *R*_aw_ in 6-year-old children. Therefore, direct comparisons with results of other groups are difficult. However, FVC can act as a surrogate of TLC because 80% of TLC is respirable and changes of TLC are reflected in changes of FVC in persons without severe respiratory illnesses such as emphysema. Therefore, qualitative and quantitative comparisons are possible. PEF and, to a smaller extent, FEV_1_ are dependent on resistance, and comparisons of effects on *R*_aw_ with effects on those lung function measures from other studies are possible in a restricted manner.

The association of annual TSPs and TLC found in our study shows similarity to the results of the Bitterfeld study in East Germany with repeated cross sections between 1992 and 1999 (Frye et al. 2003). A significant increase of 4.7% of adjusted FVC, but no effect on FEV_1_, was reported in schoolchildren 11–14 years of age, when annual TSPs decreased about 50 μg/m^3^. Transforming our results to this change of exposure resulted in an increase of 4.9% of TLC. The lack of a long-term TSP effect on *R*_aw_ in our study agreed with the lack of an effect for FEV_1_ in the Bitterfeld study.

Furthermore, the TSP-associated changes in TLC observed in our study can be compared with changes of FVC in southern California communities (Peters et al. 1999). The decrease we observed was twice the decrease in southern California: We observed a 76.7-mL decrease of TLC (corresponding to a 61.3-mL decrease of FVC) associated with a TSP change from 40 to 80 μg/m^3^, whereas for the pupils from southern California a 34.3 and 36.9 mL decrease of FVC per increase of 40 μg/m^3^ TSPs (calculated from PM_10_) was observed. This comparison is affected by different conditions such as age of children and presence of other copollutants, but exposure of PM was in a similar range of concentration, suggesting that our results and the results of the Bitterfeld study (Frye et al. 2003) might overestimate the effect of TSPs. It is likely that trend of air pollution and trend of other factors is not separable during the period of German reunification. However, the regression coefficients for the independent variable TSPs with respect to the dependent variable TLC were nearly identical for both parts of Germany. This hints at a causal effect of TSPs not confounded by trend.

## Conclusions

This study compares lung function in 6-year-old children in East and West Germany during a time of decreasing concentrations of outdoor TSPs in East Germany. The children investigated were of ethnic homogeneity but differed in lifestyle and air pollution exposure. These differences diminished during the time of observation. No other study is available comparing results from East and West Germany during this critical time of changes. TLC showed a clear association with long-term concentrations of TSPs; *R*_aw_ was affected by short-term outdoor concentrations in East Germany. Thus, the reduction in TSPs was associated with better lung function when comparing repeated cross sections in 6-year-old children. However, there was an increasing effect of traffic-related pollution in East Germany, and the favorable effects were presumably counteracted by air pollution associated with the increased traffic.

## Figures and Tables

**Table 1:** Distribution of TSPs and SO_2_ exposure (μg/m^3^) from the previous year and from the day of investigation for the children, and regression analysis of the logarithmic transformed TSPs and SO_2_ exposure for trend (10 years), region (East vs. West), and their interaction (trend East vs. trend West).

	West Germany				East Germany			MR (95% CI)^a^		
	1991	1994	1997	2000	1991	1994	1997	Trend	East/West	Trend East/trend West
TSPs from the previous year										
No.	196	282	148	307	903	493	322			
Mean	54.35	52.22	56.11	44.20	74.45	55.68	51.29	0.80* (0.67–0.96)	1.37* (1.24–1.52)	0.62* (0.48–0.80)
SD	2.69	3.07	2.45	4.73	5.37	3.44	10.80			
Min	50.00	48.00	53.00	40.00	66.00	50.00	44.00			
Max	56.00	55.00	59.00	50.00	81.00	59.00	73.00			
TSPs on day of examination										
No.	196	282	148	287	903	456	322			
Mean	51.01	53.87	45.65	49.75	127.9	68.79	49.65	0.96 (0.74–1.23)	2.98* (2.39–3.71)	0.16* (0.10–0.26)
SD	21.81	17.69	15.88	20.20	35.33	37.68	29.61			
Min	13.00	19.60	24.60	20.00	46.00	23.40	20.00			
Max	83.00	110.3	102.9	96.00	208.0	182.2	122.0			
SO_2_ from the previous year										
No.	196	282	148	307	903	493	322			
Mean	27.25	19.73	18.27	10.07	126.9	57.65	20.19	0.33* (0.26–0.43)	5.06* (3.08–8.30)	0.17* (0.10–0.31)
SD	6.94	3.80	2.90	3.40	53.23	21.23	4.63			
Min	18.00	17.00	14.00	6.00	64.00	37.00	16.00			
Max	34.00	26.00	21.00	14.00	178.0	93.00	26.00			
SO_2_ on day of examination										
No.	196	261	139	301	903	429	322			
Mean	19.81	13.15	12.21	8.75	241.5	79.05	10.24	0.46* (0.33–0.63)	20.8* (14.3–30.3)	0.02* (0.01–0.03)
SD	13.61	6.42	5.88	6.15	136.9	62.73	6.01			
Min	5.00	5.00	5.00	5.00	35.13	15.06	3.00			
Max	57.00	30.70	30.90	38.00	576.0	261.3	31.00			

Abbreviations: CI, confidence interval; Max, maximum; Min, minimum.

a MR and 95% CI for trend (10 years), region (East/West), interaction (trend East/trend West), results of linear mixed model analysis, area (exposure from the previous year), or date (exposure from the day of investigation) treated as marginal effect.

*Significant effects (*p* < 0.05).

**Table 2:** Characteristics of the study group nonasthmatic German children living at least 2 years at their place of residence.

	West Germany				East Germany		
Characteristic	1991	1994	1997	2000	1991	1994	1997
No.	196	282	148	307	903	498	322
Age [mean (years)]	6.4	6.4	6.4	6.4	6.3	6.1	6.2
Height [mean (cm)]	120.9	121.1	120.5	120.5	119.3	119.8	120.8
Male sex (%)	50.5	47.9	43.2	46.9	52.0	50.0	57.3
Birth weight < 2,400 g (%)	7.2	4.3	4.1	5.6	5.2	3.9	4.1
BMI > 18.4 kg/m^2^ (%)	7.7	8.5	10.8	12.4	6.0	7.9	9.0
Temperature < 0°C at day of examination (%)	0.0	0.0	0.0	0.0	41.0	15.4	0.0
Fossil-fuel heating at home (%)	13.1	24.3	16.4	6.8	69.4	44.5	26.1
Gas cooking at home (%)	8.3	8.9	4.8	3.9	73.0	56.7	33.5
Parental education school years ≤ 10 (%)	53.9	50.2	67.6	70.8	49.1	55.6	49.2
Bedroom sharing (%)	58.8	53.0	41.5	52.8	64.8	57.5	42.7
Tobacco smoke exposure^a^ (%)	59.3	55.6	50.7	35.9	52.9	49.5	37.6
Traffic exposure^b^ (%)	45.9	63.1	59.5	46.4	63.0	69.8	56.9

a Smoking of mother during pregnancy and/or smoking at child’s home.

b Living < 50 m from a traffic road.

**Table 3:** Lung function of nonasthmatic German children living at least 2 years at their place of residence.

	West Germany				East Germany		
	1991	1994	1997	2000	1991	1994	1997
*R*_aw_ (kPa × sec/L)							
No.	188	275	148	307	883	451	322
Minimum	0.247	0.261	0.427	0.352	0.255	0.309	0.300
25th Percentile	0.467	0.519	0.594	0.546	0.553	0.553	0.562
Median	0.554	0.594	0.673	0.626	0.651	0.634	0.641
75th Percentile	0.678	0.715	0.763	0.704	0.773	0.725	0.749
Maximum	1.008	1.465	1.352	1.015	1.710	1.286	1.267
Arithmetic mean	0.579	0.620	0.698	0.634	0.672	0.650	0.665
TLC (L)							
No.	156	188	137	257	664	373	291
Minimum	1.49	1.31	1.50	1.41	1.22	1.35	1.39
25th Percentile	1.96	2.01	1.99	2.02	1.85	1.94	2.00
Median	2.13	2.16	2.11	2.18	2.06	2.13	2.19
75th Percentile	2.35	2.39	2.30	2.38	2.24	2.36	2.39
Maximum	2.71	3.33	2.92	3.08	3.34	2.97	3.50
Arithmetic mean	2.14	2.21	2.15	2.20	2.05	2.15	2.20

**Table 4:** Influence of long-term and short-term TSP concentrations on lung function of German nonasthmatic children living at least 2 years at their residence, nonstratified analysis but with interaction terms.

		MR (95% CI)^a^					
		TSPs		Interaction with TSP daily mean			
	No.	Annual mean	Daily mean	Traffic	Region	Traffic	Region
*R*_aw_ (kPa × sec/L)	2,216	0.994 (0.957–1.033)	0.969* (0.936–1.004)	0.973* (0.965–0.981)	1.053* (1.012–1.097)	1.072* (1.056–1.089)	0.984 (0.925–1.046)
TLC (L)	1,763	0.938* (0.884–0.996)	0.996 (0.988–1.004)	1.020* (1.006–1.034)	1.012 (0.951–1.076)	0.973* (0.955–0.991)	0.996 (0.909–1.092)

a Adjusted MR and 95% CI for 40-μg/m^3^ increase of TSPs, traffic (living < vs. ≥ 50 m from a busy road), region (East vs. West German sites), and their interactions with TSPs: Results of linear mixed model analysis adjusted for education, bedroom sharing, sex, age, height, BMI, birth weight, heating with fossil fuels, cooking with gas, passive smoking and/or maternal smoking during pregnancy, outdoor temperature on the day of investigation; area treated as marginal effect.

*Significant effects (*p* < 0.1).

**Table 5:** Influence of long-term and short-term TSP concentrations on lung function of German nonasthmatic children living at least 2 years at their residence, stratified analysis [MR (95% CI) TSPs].^a^

	West			East		
	No.	Annual mean	Daily mean	No.	Annual mean	Daily mean
*R*_aw_ (kPa × sec/L)						
Traffic	416	1.111 (0.922–1.338)	0.942* (0.918–0.967)	927	0.984 (0.945–1.014)	0.996 (0.976–1.016)
Nontraffic	343	1.096 (0.966–1.243)	0.982 (0.938–1.028)	530	0.967 (0.901–1.038)	1.025* (0.999–1.051)
TLC (L)						
Traffic	328	0.973 (0.913–1.038)	0.981* (0.971–0.992)	724	0.969 (0.930–1.009)	0.998 (0.993–1.003)
Nontraffic	278	0.930* (0.875–0.988)	1.007 (0.976–1.040)	433	0.938* (0.910–0.967)	0.999 (0.985–1.012)

a Adjusted MR and 95% CI for 40-μg/m^3^ increase of TSPs: Results of linear mixed model analysis, adjusted for education, bedroom sharing, sex, age, height, BMI, birth weight, heating with fossil fuels, cooking with gas, passive smoking and/or maternal smoking during pregnancy, outdoor temperature on the day of investigation; area treated as marginal effect.

*Significant effects (*p* < 0.1).

**Table 6:** Influence of living near a busy road on lung function of German nonasthmatic children living at least 2 years at their residence.^a^

	West		East		East 1994/1997	
	No.	MR (95% CI)	No.	MR (95% CI)	No.	MR (95% CI)
*R*_aw_ (kPa × sec/L)	770	1.019* (1.003–1.035)	1,488	1.007 (0.989–1.025)	363	1.021* (1.005–1.037)
TLC (L)	617	0.994* (0.991–0.998)	1,182	1.006 (0.998–1.014)	306	0.989* (0.986–0.992)

a Adjusted MR and 95% CI for living < versus ≥ 50 m away from a busy road: results of linear mixed model analysis, adjusted for education, bedroom sharing, sex, age, height, BMI, birth weight, heating with fossil fuels, cooking with gas, passive smoking and/or maternal smoking during pregnancy, outdoor temperature on the day of investigation; area treated as marginal effect.

*Significant effects (*p* < 0.1).
